# Multi-Influenza HA Subtype Protection of Ferrets Vaccinated with an N1 COBRA-Based Neuraminidase

**DOI:** 10.3390/v15010184

**Published:** 2023-01-09

**Authors:** Amanda L. Skarlupka, Xiaojian Zhang, Uriel Blas-Machado, Spencer F. Sumner, Ted M. Ross

**Affiliations:** 1 Center for Vaccines and Immunology, University of Georgia, Athens, GA 30602, USA; 2 Athens Veterinary Diagnostic Laboratory, University of Georgia, Athens, GA 30602, USA; 3 Department of Pathology, University of Georgia, Athens, GA 30602, USA; 4 Department of Infectious Diseases, University of Georgia, Athens, GA 30602, USA; 5 Florida Research and Innovation Center, Cleveland Clinic, Port Saint Lucie, FL 34987, USA; 6 Department of Infection Biology, Lehner Research Institute, Cleveland Clinic, Cleveland, OH 44106, USA

**Keywords:** influenza, ferrets, vaccination, viral transmission, neuraminidase, N1, COBRA

## Abstract

The influenza neuraminidase (NA) is a promising target for next-generation vaccines. Protection induced by vaccination with the computationally optimized broadly reactive NA antigen (N1-I COBRA NA) was characterized in both influenza serologically naive and pre-immune ferret models following H1N1 (A/California/07/2009, CA/09) or H5N1 (A/Vietnam/1203/2004, Viet/04) influenza challenges. The N1-I COBRA NA vaccine elicited antibodies with neutralizing ELLA activity against both seasonal and pandemic H1N1 influenza, as well as the H5N1 influenza virus. In both models, N1-I COBRA NA-vaccinated ferrets that were challenged with CA/09 virus had similar morbidity (weight loss and clinical symptoms) as ferrets vaccinated with the CA/09 HA control vaccine. There were significantly reduced viral titers compared to the mock-vaccinated control animals. Ferrets vaccinated with N1-I COBRA NA or Viet/04 NA vaccines were protected against the H5N1 virus infection with minimal clinical symptoms and negligible weight loss. In contrast, ferrets vaccinated with the CA/09 NA vaccine lost ~10% of their original body weight with 25% mortality. Vaccination with either HA or NA vaccines did not inhibit contact transmission of CA/09 virus to naïve cage mates. Overall, the N1-I COBRA vaccine elicited protective immune responses against both H1N1 and H5N1 infections and partially mitigated disease in contact-transmission receiving ferrets. These results indicate that the N1-I COBRA NA performed similarly to the CA/09 HA and NA positive controls. Therefore, the N1-I COBRA NA alone induces protection against viruses from both H5N1 and H1N1 subtypes, indicating its value as a vaccine component in broadly protective influenza vaccines.

## 1. Introduction

Influenza viruses, type A and B, circulate pervasively in the global human population and upon infection, induce a contagious upper respiratory illness. Influenza virus infection often results in fatigue, fever, sneezing, body aches, nausea, and, in severe cases, pneumonia and death. Type A influenza viruses has a broad host species range, whereas Type B influenza viruses are mainly human isolated. Avian, swine, and other host species act as reservoirs for zoonotic transmission and lead to constant reintroduction of influenza viruses with pandemic potential due to individuals lacking pre-existing immunity to novel strains. The two major surface proteins, hemagglutinin (HA) and neuraminidase (NA), classify the influenza A viruses into subtypes. The sequentially numbered protein subtypes denote antigenically distinct groups. In humans, the H1N1 and H3N2 viral subtypes co-circulate seasonally with occasional zoonotic spillover infections, most commonly with avian-origin H5N1 and H7N9 viruses [1]. Broadly protective influenza virus vaccines are currently unavailable for seasonal human influenza or zoonotic pandemic viral variants [2]. Governmental agencies prioritized the funding of the development of such a vaccine in 2019 [3,4].

Influenza viruses transmit, primarily, through airborne transmission or direct contact with infectious individuals and surfaces. Ideally, an effective influenza virus vaccine will prevent infection and prevent transmission to another person. Vaccination can also lower viral shedding from virally exposed vaccinated individuals by either reducing the peak viral load or decreasing the shedding timeframe [5]. Currently, split-inactivated vaccines are non-sterilizing and infection-permissive, but vaccination reduces disease symptoms and adverse outcomes following infection [6,7]. 

Influenza virus vaccine development has often overlooked the influenza virus neuraminidase as a potential vaccine candidate antigen. Split-inactivated vaccines are standardized based upon HA content and are not quantified or standardized for NA content. The immunodominance of the HA further dampens the immune response to NA [8,9]. The HA protein of the virus then uses sialic acid receptors to mediate entry into cells, whereas the NA protein cleaves the sialic acid receptors. This function improves viral motility, allows the release nascent virions from host cells and prevents self-aggregation [10]. Anti-NA polyclonal and monoclonal antibodies protect mice and ferrets from influenza virus infection [11,12,13]. NA-inhibiting antibodies decrease influenza virus disease severity; vaccine effectiveness can be enhanced through the synergy of NA and HA inhibiting antibodies [4,14,15]. 

The goal of the current study was to evaluate a next-generation neuraminidase vaccine based upon computationally optimized broadly reactive antigen (COBRA) methodology in ferrets [16,17]. The NA antigen was designed for the N1 influenza subtype, designated N1-I [16,17]. This COBRA NA antigen elicited inhibitory antibody responses to a panel of HxN1 viruses encompassing all three genetic lineages of N1: N1.1 (avian; human pandemic), N1.2 (human seasonal), and N1.3 (classical swine). In contrast, wildtype N1.1 and N1.3 antigens elicited cross-reactive NAI antibodies among the lineages, but could not inhibit the NA of H1N1 N1.2 viruses. Likewise, the antisera to the N1.2 NA did not inhibit the N1.1 or N1.3 clade viruses. Previously, our group demonstrated that mice vaccinated with a recombinant N1-I COBRA NA protein were protected against viral influenza. The infection results of the N1-I COBRA NA-vaccinated groups and homologous vaccine groups were similar. Further, the N1-I COBRA NA groups maintained lower viral titers than the mock-vaccinated animals. Consequently, the protective efficacy of the NA COBRA vaccine was quantified in the ferret model. 

The ferret model is the gold standard for vaccine efficacy testing due to its natural susceptibility to human influenza, the ferret’s sizeable respiratory system, and similar immunological and physiological responses to vaccination and infection [18]. The ferret is an excellent model for pre-immunity studies that more closely model human infection due to humans experiencing immune imprinting from previous viral infections [19]. In this study, we tested the ability of the N1-I COBRA NA to elicit protective immune responses in a naïve and H1N1 pre-immune ferret model. The N1-I COBRA NA results were compared to ferrets vaccinated with homologous and heterologous wildtype NA and HA antigens following challenges with A/California/07/2009 (H1N1), A/Vietnam/1203/2004 (H5N1), or A/Brisbane/59/2007 (H1N1). Vaccines were evaluated for elicitation of broadly reactive antibodies, protection against both morbidity and mortality, and the inhibition of viral transmission between ferrets.

## 2. Materials and Methods

### 2.1. Viruses

The historical influenza virus A/Singapore/06/1986 (Sing/86; H1N1; BSL-2) was used for establishing pre-immunity, while A/California/07/2009 (CA/09; H1N1; BSL-2), A/Vietnam/1203/2004 (Viet/04; H5N1; BSL-3 select agent), and A/Brisbane/59/2007 (Bris/07; H1N1; BSL-2) were used for infections. In addition to Sing/86 and CA/09, the following viruses were used in the hemagglutinin inhibition assay (HAI) and enzyme-linked lectin assay (ELLA): A/Vietnam/1203/2004 PR8 reassortant (Viet/04xPR8; H5N1; BSL-2; 6:2 reassortant virus with A/Puerto Rico/8/1934 internal genes and Viet/04 HA and NA gene segments), Bris/07, and A/swine/North Carolina/154704/2015 (Sw/NC/15; H1N1; BSL-2). Sw/NC/15 was propagated in Madin–Darby canine kidney (MDCK) cells; all other viruses were propagated in specific pathogen-free (SPF) 10-day-old embryonated chicken eggs. MDCK cells were maintained with Dulbecco’s modified Eagle’s medium (DMEM) with 10% heat-inactivated fetal bovine serum (FBS) with 1% penicillin-streptomycin (P/S) at 37 °C with 5% CO_2_.

### 2.2. Vaccines

Recombinant soluble proteins used for vaccination included: CA/09 NA, Viet/04 NA, Bris/07 NA, N1-I COBRA NA, and CA/09 HA. The optimized coding sequences for wildtype and COBRA proteins in pcDNA3.3 vectors were expressed in soluble proteins using a HEK-293T cell expression line, as described previously [16,20]. Proteins were extracted using HisTrapExcel columns with the AKTA Pure System (GE Healthcare Bio-Sciences AB, Uppsala, Sweden). Purified proteins were concentrated with phosphate-buffered saline + 0.1% *w/v* sodium azide (PBSA). Protein concentration was determined using Micro BCA Protein Assay Reagent kits (Pierce Biotechnology, Rockford, IL, USA), and aliquots of each protein were stored at −80 °C until used for vaccination. To assess purity, 1 μg of each NA protein sample was mixed 3:1 with 4 × Laemmli Sample Buffer (Bio-Rad, Hercules, CA, USA) and subjected to sodium dodecyl sulfate-polyacrylamide gel electrophoresis (SDS-PAGE). The precast 10% SDS gel (Thermo Fisher Scientific, Waltham, MA, USA) with loaded NA protein samples were electrophoresed at 200 V for 30 min and then stained with PageBlue Protein Staining Solution (Thermo Scientific) for 1 h at room temperature and de-stained with distilled water to visualize the protein bands.

### 2.3. Animals

Female Fitch ferrets (*Mustela putorius furo*) between 6 and 15 months of age were sourced from Triple F Farms (Gillett, PA, USA) after de-scenting and spaying. Each animal was confirmed to be serologically naive to the A/California/07/2009 H1N1 influenza virus with sera collected prior to vaccination or pre-immune infection. When not infected, ferrets were pair housed with free access to food, water, and enrichment. Ferrets were anesthetized with vaporized isoflurane before bleeds, vaccination, infection, nasal washes, and euthanasia. All animal procedures were performed following the Guide for the Care and Use of Laboratory Animals, Animal Welfare Act, and Biosafety in Microbiological and Biomedical Laboratories (AUP: A2020 11-016-Y1-A6).

**Figure 1:**
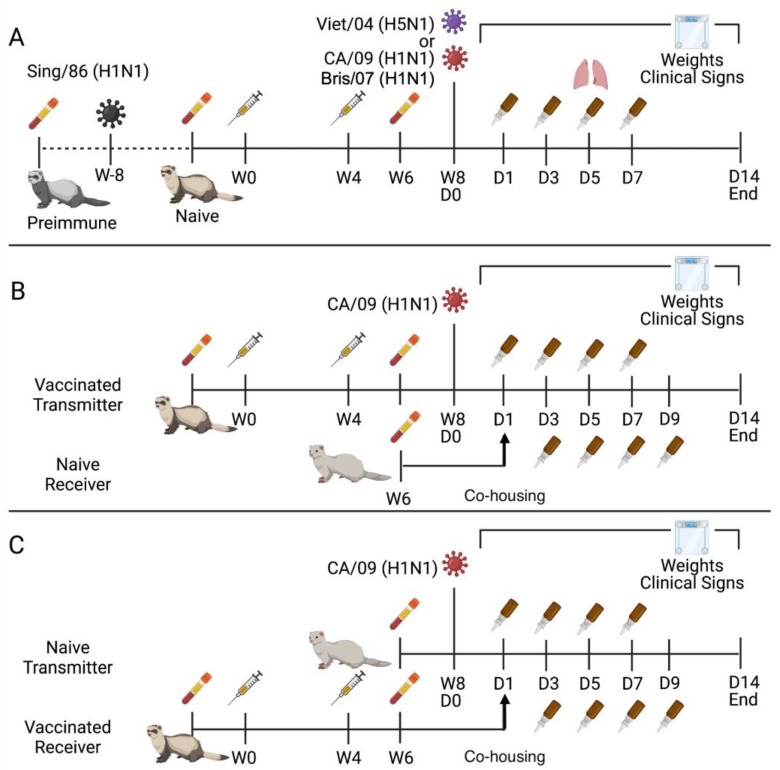
Naïve, pre-immune, and contact transmission ferret model design. Naïve or pre-immune ferrets were vaccinated with 15 μg of adjuvanted recombinant protein in a prime-boost schema after pre-bleeding and pre-immunization if necessary (**A**). Animals were challenged with either Viet/04 or CA/09. On day 5 p.i., four animals were harvested for lung viral titers and histopathology for CA/09 challenge. Contact transmission between vaccinated transmitters and influenza naïve receivers was studied by vaccinating the transmitter with 15 μg recombinant protein with adjuvant in a prime-boost, infecting with CA/09, and on day 1 p.i. co-housing the intranasally infected ferret with the naïve receiver after nasal wash (**B**). Contact transmission between influenza naïve infected transmitters and vaccinated receivers was similar, except the naïve ferret was infected and then the vaccinated ferret was co-housed on day 1 p.i. (**C**). Weights and clinical signs were recorded for all ferrets for a maximum of 14 days post-influenza exposure. Nasal washes were harvested on days 1, 3, 5, and 7 p.i. for intranasally infected animals and on days 3, 5, 7, and 9 p.i. relative to the day of infection for the transmitting ferret for the contact transmission animals.

Ferrets were made pre-immune by infecting the naïve ferrets (serologically naive to CA/09 influenza virus) intranasally with the Sing/86 influenza virus 8 weeks prior to initial vaccination. Ferrets (pre-immune or naïve) were vaccinated intramuscularly in the thigh muscle with 15 μg of protein in a total volume of 500 μL (Figure 1A). Addavax adjuvant (InvivoGen, San Diego, CA, USA) was mixed in a 1:1 ratio (250 μL sterile PBS with protein: 250 μL Addavax). Mock-vaccinated groups received 250 μL of sterile PBS with 250 μL of Addavax. Four weeks from the prime vaccination, the animals received a booster vaccine of the same mixture. At least two weeks after the boost, blood was collected in BD Vacutainer SST tubes. After 30 min at room temperature (RT), serum was separated by processing the tubes at 2500 rpm for 10 min. Purified serum was stored at −20 °C until analysis.

### 2.4. Direct and Contact Transmission Ferret Infections

Ferrets were directly infected either to establish pre-immunity before vaccination (Sing/86) or to challenge the vaccine groups for protection characteristics (CA/09, Viet/04, and Bris/07). Direct infection was performed intranasally with 1 mL total volume with 500 μL administered to each naris. The infection dose used for Sing/86, CA/09, and Bris/07 was 1 × 10^6^ plaque-forming units (PFU)/mL. Whereas the infection dose used for Viet/04 was 1 × 10^5^ PFU/mL. After infection, animals were observed twice daily for clinical signs and weighed once daily until two consecutive days without signs. On days 1, 3, 5, and 7 post-infection (p.i.) nasal washes were performed with 3 mL of sterile PBS.

Groups of influenza-naïve ferrets were primed and boosted with HA or NA vaccines. Approximately 4 weeks after the booster, two types of models were used to evaluate the virus transmission between vaccinated animals and unvaccinated animals: (i) the vaccinated ferrets (Transmitter) were infected with CA/09 influenza virus intranasally, and unvaccinated influenza-naïve ferrets (Receiver) were introduced 1-day p.i. (Figure 1B). (ii) The vaccinated ferrets (Receiver) were exposed to the virus by co-housing with a donor, unvaccinated influenza-naïve ferrets (Transmitter), that had been infected with CA/09 one day previously (Figure 1C). The receiving ferret was placed with the directly infected ferret on day 1 p.i. after nasal wash to evaluate contact transmission. The receiving ferret remained pair housed with the transmitting ferret until the end of the observation period. The receiving ferret was nasal washed on days 3, 5, 7, and 9 p.i. of the transmitting ferret, i.e., days 2, 4, 6, and 8 post-contact. All nasal wash samples were stored at −80 °C until viral titration.

When a cumulative clinical score of three was reached, the animal was humanely euthanized. Clinical signs with their scores were as follows: nasal discharge/sneezing/diarrhea (0.5; not used for humane endpoint calculation but used for graphical representation), lethargy (1), dyspnea (2), cyanosis (2), neurological signs (3), moribund (3), laterally recumbent (3), failure to respond to stimuli (3), weight loss of 20–25% (2), and weight loss of greater than 25% (3). The maximum of the two clinical scores recorded for each day was used for analysis.

### 2.5. Histopathological Analysis

Histopathological samples were collected from designated animals prior to infection and were euthanized with B-euthanasia on day 5 p.i. The left cranial and caudal lobes were sectioned into quarters, placed on dry ice, and stored at −80 °C until viral titration. The right cranial, middle, caudal lobe, and accessory lobe were infused intratracheally with neutral-buffered, 10% formalin fixative solution (BF). The trachea and right lung were extracted and placed in BF. The submandibular lymph node was extracted and set in BF. The head was removed at the junction of the cricoid cartilage and tracheal rings. The nasal cavity was fixed with BF administration through the nasopharynx until BF drained from both nares. All samples were stored in BF for one week, after which 70% ethanol solution replaced the BF. The skull was decalcified in Kristensen’s solution for two weeks. All tissues were embedded in paraffin, and sectioned as follows: coronal sections through the nasal cavity, transverse sections through the middle ear, and cross sections through the submandibular lymph node, trachea, and right lung lobes. The 5 μm thick sections were stained with hematoxylin and eosin (H&E). To identify T-cells in the submandibular lymph nodes, CD3 immunohistochemistry for T-cells (polyclonal rabbit anti-CD3 antibody (Dako A0452) was performed. 

The microscopic exam consisted of the evaluation of the nasal cavity (at 9 levels), ear (middle), trachea, and the right lung lobes (cranial, middle, and caudal) for the presence or absence of inflammation. Microscopically, lesion (tissue change or alteration) incidence, severity, and distribution were recorded. If absent (i.e., histologically normal), a score of 0 was assigned. If present, the severity of the lesions was recorded as minimal, mild, moderate, or severe, with severity scores of 1 through 4, respectively, based on an increasing extent and/or complexity of change, unless otherwise specified. Lesion distribution was recorded as focal, multifocal, or diffuse, with distribution scores of 1, 2, or 3, respectively. A group histopathological score was calculated by adding individual animal severity and distribution scores. All histopathological work was conducted in the spirit of the US FDA Good Laboratory Practice regulations (21 CFR Part 58 and subsequent amendments) and all microscopic evaluations were performed on the H&E-stained sections by a board-certified pathologist (UBM).

### 2.6. Influenza Virus Plaque Assay

The nasal wash and lung samples were processed for viral titration. The nasal wash samples were diluted in 10-fold serial dilutions in DMEM + P/S before addition to the cells. The lung samples were weighed and then homogenized in a corresponding quantity of DMEM + P/S such that 0.1 g was resuspended in 1 mL DMEM + P/S. The homogenized lung was passed through a. 0.70 μm nylon filter (Corning Cell Strainer, Sigma Aldrich, St. Louis, MO, USA). The filtrate was then diluted in 10-fold serial dilutions in DMEM + P/S before addition to the cells. The upper right quadrant of the left cranial lobe and the lower left quadrant of the left caudal lobe were processed for viral lung titers.

MDCK cells were seeded at 2.5 × 10^5^ cells per well of a 12-well tissue-culture treated plate. The next day the confluent cells were washed with DMEM + P/S and overlaid with 100 μL of the viral sample. Plates were incubated at RT with shaking every 15 min. The cells were then washed with DMEM + P/S and overlaid with 1 mL of plaque medium (minimum essential media with P/S, 2 mM L-glutamine,1.5 mg/mL NaHCO_3_, 10 mM HEPES, 5 μg/mL Gentamycin, and 1.2% Avicel RC-591 NF (MFC corporation, Philadelphia, PA, USA). For Viet/04 virus, trypsin was not added, but for CA/09 virus, 1.5 μg/mL TPCK-treated trypsin was added (Sigma-Aldrich). Plates were incubated at 37 °C with 5% CO_2_. After 48 h (Viet/04) or 72 h (CA/09), plates were removed, washed with PBS, and fixed with BF for 15 min. Afterwards, the plaques were visualized by staining with 1% crystal-violet for 10 min. The plaques were counted and back-calculated to determine the PFU/mL for nasal wash viral titers and the PFU/g for lung tissue viral titers. All plaques were conducted in duplicate for each sample, and the average value was taken for analysis. The limit of detection of nasal wash and viral lung titers were 1.0 log_10_(PFU/mL) and 2.0 log_10_(PFU/g). The limit of quantification was defined as greater than or equal to 10 countable plaques, which led to reliable lower limits of 2.0 log_10_(PFU/mL) and 3.0 log_10_(PFU/g) for nasal wash and viral lung titer values, respectively.

### 2.7. Hemagglutination Inhibition (HAI) Assay

Ferret sera were treated with three parts receptor destroying enzyme (RDE, DENKA SEIKEN, Tokyo, Japan). Sera and RDE were incubated at 37 °C for 18–20 h and then heat-inactivated at 56 °C for 1 h. After reaching RT, six parts PBS was added to the samples. The HAI assay was performed as previously described. The H1N1 viruses and Viet/04xPR8 virus were adjusted to 1:8 HA units/50 μL with 0.8% turkey erythrocytes (Lampire Biologicals, Pipersville, PA, USA) and 1% horse erythrocytes (Lampire Biologicals, Pipersville, PA, USA), respectively. The serum was diluted two-fold in V-bottom 96 well plates and incubated in equal volume with the virus for 20 min at RT. After which, an equal volume of the respective erythrocytes was added. After 30 min for H1N1 viruses and 60 min for Viet/04xPR8 H5N1 virus, the plates were tilted, and the reciprocal dilution of the last well to not be agglutinated was recorded as the HAI titer. The last column of the plate contained no sera—only PBS, virus, and erythrocytes—served as the negative control.

### 2.8. Neuraminidase Inhibition Assay (NAI); Enzyme-Linked Lectin Assay (ELLA)

Sera treatment for the ELLA assay was similar to the treatment for the HAI assay, except heat inactivation was performed for 8 h to completely deactivate the NA activity of the *Vibrio cholerae* neuraminidase. The NA activity of the virus was determined as previously described and diluted to a concentration providing 90–95% NA activity [21]. From an initial dilution of 1:100, sera were diluted two-fold in Dulbecco’s phosphate-buffered saline containing 0.133 g/L CaCl_2_ and 0.1 g/L MgCl_2_ (DPBS), 1% BSA, and 0.5% Tween-20 (DPBS-BT). The sera were added to a PBS + Tween-20 (PBS-T) washed fetuin plated coated previously overnight with 100 μL of 25 μg/mL fetuin. The serial dilutions were added in 25 μL in duplicate per ferret sera sample. In the control wells, 50 μL of DPBS-BT was added in substitution of sera. The control wells included at least six wells with no sera and no virus for the subtraction of the background absorbance and another minimum of six wells with no sera and only virus to serve as the 100% NA activity threshold. The diluted virus was added in 50 μL, and the plate was rocked to mix. Plates were incubated at 37 °C with 5% CO_2_ for 16–18 h. After which, they were washed 6X with PBS-T, and 100 μL of peanut agglutinin-HRPO (Sigma-Aldrich, St. Louis, MO, USA) was added at a dilution of 1:1000 in DPBS-T. Plates were incubated in the dark for 2 h at RT. After washing 3X in PBS-T, 100 μL of o-phenylenediamine dihydrochloride (OPD; Sigma-Aldrich, St. Louis, MO, USA) in 0.05 M phosphate-citrate buffer with 0.03% sodium perborate pH 5.0 (Sigma-Aldrich, St. Louis, MO, USA) was added to the plates. They were incubated in the dark at RT for 10 min and stopped with 100 μL of 1 N sulfuric acid. The absorbance was read at 490 nm using a spectrophotometer (PowerWave XS; BioTek, Winooski, VT, USA). The background absorbance was subtracted, and the serum-containing wells were normalized with the average of the virus-only wells defining 100% NA activity. Non-linear regression was conducted in Prism 9.1 using the duplicates to provide the average estimated log_10_ 50% NI titer for individual ferrets.

### 2.9. Statistical Analysis

The data were analyzed by either a two-way ANOVA or a REML mixed effects model if data were missing. Repeated measures were used to account for the ferret variation. Initially, the interactions were fit between the two main effects (usually vaccine group and day p.i.). If the interaction was not significant with an F-test, the analysis was then conducted with only the main effects. Tukey’s multiple comparison test was conducted first. If there was no significant difference between the vaccinated groups to each other, a Dunnett’s multiple comparison test was conducted using the mock-vaccinated as the control group. Survival curves were analyzed using the log-rank Mantel–Cox test with asymmetrical 95% confidence intervals. The mean value with standard deviation error bars were depicted on all figures except for clinical scores. Clinical score figures depicted the standard error of the mean, with the individual values shown in the background. The offsetting values of the weight loss and viral nasal wash titers were determined by adjusting the days in increments of one, until the mock-vaccinated groups were visually aligned with each other. All the statistical data are available in the Supplementary Materials.

## 3. Results

### 3.1. N1-I COBRA NA Mitigated Clinical Signs and Viral Titers in Naïve Ferrets Directly Challenged with CA/09 H1N1

**Figure 2:**
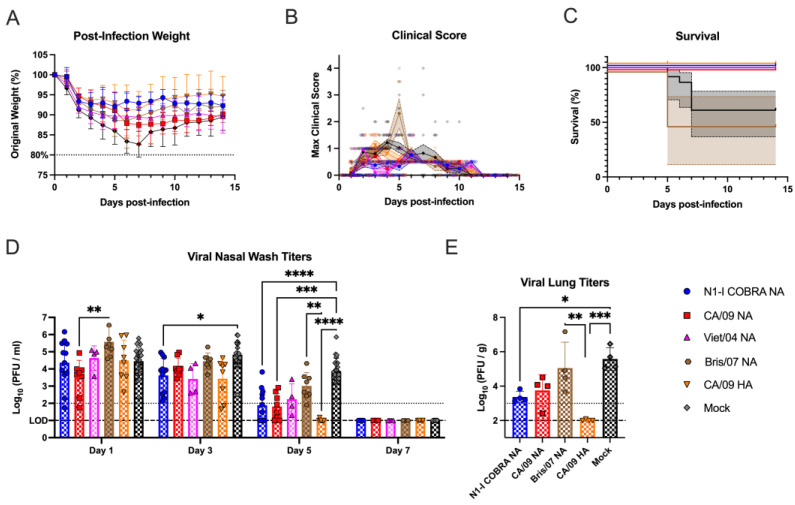
N1-I COBRA NA vaccines performed similarly to CA/09 HA and NA vaccines in the naïve ferret model intranasally challenged with CA/09 H1N1. (**A**) After infection with CA/09, the weight during the challenge was normalized to the percentage of the original starting weight before infection. (**B**) The maximum clinical scores from the morning and evening checks were displayed with a score of three or greater indicating clinical endpoint. Individual ferret clinical scores are shown with the line connecting the mean score and the range of the standard error of the mean shaded. (**C**) Survival was determined after accounting for censored histopathology ferrets purposefully euthanized on day 5 p.i. The 95% asymmetrical confidence intervals are shown as shadow for the groups that did not have 100% survival. (**D**) The viral nasal wash titers for all vaccinated groups were determined through plaque assay and analyzed with a mixed effects model. (**E**) The viral lung titers were quantified by plaquing the homogenized tissue from both the upper and lower left lung lobes. The lobe location was not a significant factor, and, thus, the titers were analyzed with a one-way ANOVA. Viral lung titers were not harvested for the Viet/04 NA group. The limit of detection (LOD; dashed line) was 1.0 log_10_(PFU/mL) and 2.0 log_10_(PFU/g) for the nasal wash titers and lung titers, respectively. The limit of quantification (dotted line) was 2.0 log_10_(PFU/mL) and 3.0 log_10_(PFU/g) for the nasal wash titers and lung titers, respectively. Tukey’s test for multiple comparisons was used for viral titer comparisons. All error bars represent standard deviation. The minimum weight before clinical endpoint was 75%, with 80% denoting an increase in clinical scoring. Adjusted *p*-value: * ≤ 0.05, ** ≤ 0.01, *** ≤ 0.001, **** ≤ 0.0001.

Naïve ferrets were vaccinated with the N1-I COBRA NA or one of the wild-type NA or HA vaccines, and then challenged with the CA/09 virus (Figure 1A). After challenge, the mock-vaccinated ferrets lost 15–20% of their body weight by day 7 p.i. (Figure 2A). In contrast, ferrets vaccinated with N1-I COBRA NA lost significantly less weight (5–7% of their original body weight) by day 3 p.i. (adj. *p*-value < 0.05) and then maintained that weight for the remainder of the study (Figure 2A). This weight loss was similar to ferrets vaccinated with CA/09 HA, and less than those ferrets vaccinated with wild-type NA vaccines (Figure 2A). Higher clinical scores and morbidity were observed for ferrets with greater weight loss (Figure 2B,C). As an example, by day 2 p.i., ferrets vaccinated with N1-I COBRA NA or CA/09 NA vaccines had lower scores than ferrets vaccinated with the Viet/04 NA group. At day 4 p.i., mock-vaccinated ferrets had greater clinical scores than the N1-I COBRA NA, Viet/04 NA, and CA/09 NA-vaccinated ferrets (Figure 2B). Four of the eight mock-vaccinated ferrets had high clinical scores, reached the clinical endpoint, and were sacrificed by day 5 p.i. (Figure 2C). The Bris/07 NA and mock-vaccinated groups exhibited 50% and 60% survival by the end of the observation period, respectively. All other vaccinated ferrets maintained 100% survival. 

Over the course of infection, nasal wash samples were taken every other day to quantify viral titer in the upper respiratory tract (Figure 2D). Virus was recovered from all vaccinated ferrets at day 1 p.i. and were not detectable by day 7 p.i. On day 3 p.i., N1-I COBRA NA-vaccinated ferrets had significantly lower viral titers than the mock group. By day 5 p.i., the CA/09 HA, N1-I COBRA NA, and CA/09 NA-vaccinated ferrets had viral titers significantly lower than the mock-vaccinated ferrets, and at or near the limit of detection (1.0 log_10_(PFU/mL)). The viral shedding for ferrets in Bris/07 NA and Viet/04 NA groups were not significantly different from the ferrets in mock group, with the Bris/07 NA-vaccinated ferrets having a higher mean viral shedding titer on day 5 p.i. than Viet/04 NA-vaccinated ferrets. These results were corroborated by the viral lung titers of the left lung lobes collected on day 5 p.i. (Figure 2E). The viral lung titers for the ferrets in CA/09 HA group was below the limit of detection (2.0 log_10_(PFU/g lung tissue)). The ferrets in N1-I COBRA NA group had significantly lower viral loads in the lung tissue compared to the ferrets in mock group (adj. *p*-value = 0.0167). The ferrets in Bris/07 NA (antigenically distinct to CA/09 NA) group was not significantly different for viral loads from the ferrets in mock group but had a greater mean titer than the ferrets in CA/09 HA group. Again, the N1-I COBRA NA performed similarly to the CA/09 positive controls.

**Figure 3:**
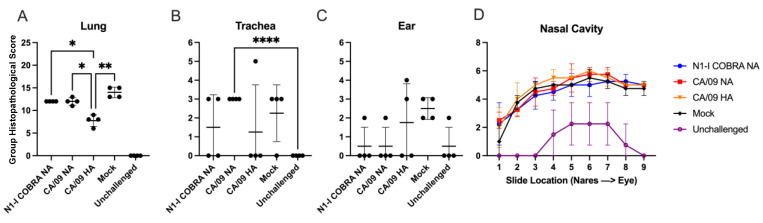
N1-I COBRA NA vaccine induced similar inflammation in the upper and lower respiratory tract as the CA/09 HA and CA/09 NA vaccines in naïve ferret model after intranasal infection with CA/09 H1N1. H&E-stained formalin-fixed and embedded tissues were scored for the severity and distribution of inflammation. (**A**) The NA-based vaccines had the same mean severity score and were significantly greater than the CA/09 HA vaccine group. All groups were significantly different (*p* < 0.01) compared to the unchallenged group (not shown). (**B**) The trachea was examined for inflammation and the CA/09 NA-vaccinated group had higher incidence and mean severity scores compared to all other groups. (**C**) The inflammation in the middle and inner ear was mild. The mock group had 100% incidence. (**D**) Sections of the nasal cavity from the nares to the eye were examined for inflammation. All challenged animals had similar group histopathological scores compared to the unchallenged group, in which the histopathological score was representative of background, non-significant inflammatory cellular infiltrates. A two-way repeated measures ANOVA with Tukey’s test for multiple comparisons was used for statistical analysis of the lung, trachea, and ear sections, and the same analysis conducted separately for the nasal cavity sections. Adjusted *p*-value: * ≤ 0.05, ** ≤ 0.01, **** ≤ 0.0001.

In addition to lung viral titers, histopathological samples were analyzed at day 5 p.i. (Figure 3). Of all tissues examined, significant tissue alterations consisted of tissue inflammation, which was most consistently observed in the lungs, followed by the nose. The severity and distribution of the inflammation in the right lung was significantly greater in all vaccine groups compared to the unchallenged unvaccinated control group (Figure 3A). The inflamed lung tissue was hyperemic and hypercellular about the airways (bronchi and bronchioles), respiratory (or terminal) bronchioles, and blood vessels. In the most severe cases, the terminal bronchioles had necrosis and sloughing of the mucosa, with hypertrophy and hyperplasia of the adjacent mucosal epithelia. The sloughed, necrotic epithelia extended into the adjacent alveoli, which contained foamy macrophages. In the adjacent blood vessels, mild to moderate collections of lymphocytes obscured and expanded the perivascular tissues and extended into the adjacent alveolar and terminal bronchiolar interstitial tissue. In the larger bronchioles, the mucosa was thickened and hypercellular, with loss of cilia. Within a loose fibrocollagen tissue stroma, lymphocytic, plasmacytic, and neutrophilic infiltrates obscured and expanded the peribronchiolar and peribronchial tissues; infiltrates often extended into the lumens through the mucosal walls and peripherally into and between the bronchiolar smooth muscles, peribronchiolar and bronchial blood vessels, and spaced bronchiolar glands. The lumens of the larger bronchioles and bronchi contained sloughed necrotic cells, neutrophils, necrotic debris, and proteinaceous material mixed with mucus.

Of the vaccine groups, the CA/09 HA had significantly less inflammation compared to all NA vaccine groups. Comparing the degree of inflammation of the N1-I COBRA and CA/09 NA groups, they had the same group histopathological score, which was lower than that of the mock-vaccinated although insignificantly. Inflammation was observed in the lung of all challenged animals. Inflammation in the trachea was more variable (Figure 3B). The CA/09 NA group had a greater inflammation score than the unchallenged animals, and 100% of the animals having signs of inflammation. The mock challenged animals had similar inflammation levels with 75% of the animals having inflammation in the trachea. Comparatively, N1-I COBRA NA and CA/09 HA vaccine groups had an inflammation incidence of 50% and 25%, respectively. The middle/inner ear was similar to the trachea, with the prevalence of inflammation varying between groups (Figure 3C). N1-I COBRA NA, CA/09 NA and unchallenged animals had the similar profile of 25% prevalence with one animal exhibiting a score of two. The CA/09 HA-vaccinated group had 50% prevalence with two severity scores of three and four. The mock-vaccinated animals had 100% prevalence of inflammation with a mean of 2.5 severity score. There were no statistically significant comparisons between the middle/inner ear samples. Of the nasal cavity sections, the unchallenged animals had a low level of inflammation further in the nasal cavity between sections three to eight (Figure 3D). The inflamed nose tissue was diffusely hyperemic and hypercellular. Large number of neutrophils and macrophages, mixed with debris and embedded in basophilic mucinous material filled the nasal meatus and covered the mucosa. In some areas, there was loss of ciliated epithelia and replacement with low cuboidal to squamous epithelia. Neutrophils expanded the loose tissues of the underlying lamina propria and often extended into the overlying mucosa (leukocytic exocytosis). Neutrophil, macrophage, and lymphocytic infiltrates extended along the loosely arranged lamina propria/submucosa between the spaced nasal glands and dilated lymphatics and blood-engorged vessels. For all challenged animals, regardless of vaccine group, the inflammation was greater than the unchallenged, but no different when compared to each other. The submandibular lymph node had no inflammation, lymphocyte necrosis, or lymphoid depletion across all groups. The T-cell populations were investigated through immunohistochemistry. The lymphoid hyperplasia of the CD3+ cells were elevated in all challenged groups when compared to the unchallenged, but not different among each other.

### 3.2. N1-I COBRA NA Mitigated Clinical Signs and Viral Nasal Wash Titers in H1N1 Pre-Immune Ferrets Directly Challenged with CA/09 H1N1

**Figure 4:**
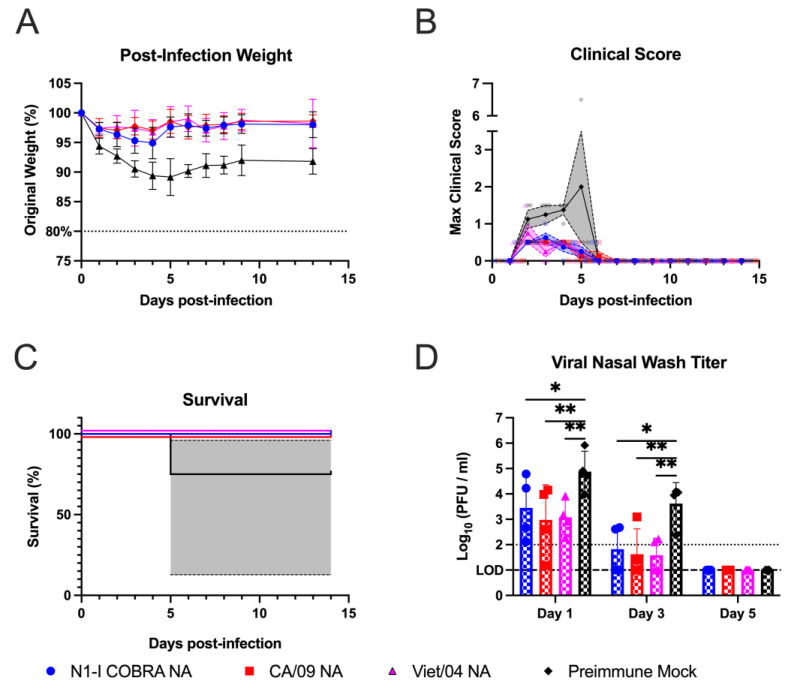
NA-vaccinated ferrets pre-immunized with Sing/86 H1N1 exhibited minimal clinical signs after intranasal infection with CA/09 H1N1. (**A**) After infection with CA/09, the weight during infection was normalized to the percentage of the original starting weight before infection. (**B**) The maximum clinical scores from the morning and evening checks were displayed with a score of three or greater indicating clinical endpoint. Individual ferret clinical scores are shown with the line connecting the mean score and the range of the standard error of the mean shaded. (**C**) The survival percentage for each group was determined. The 95% asymmetrical confidence intervals are shown as shadow for the groups that did not have 100% survival. (**D**) The nasal wash titers for all vaccinated groups were determined through plaque assay. The limit of detection (LOD; dashed line) was 1.0 log_10_(PFU/mL), and the limit of quantification (dotted line) was 2.0 log_10_(PFU/mL). A two-way repeated measures ANOVA and Dunnett’s test for multiple comparisons was used for statistical analysis with the mock-vaccinated as the control group. All error bars represent standard deviation. The minimum weight before clinical endpoint was 75%, with 80% denoting an increase in clinical scoring. Adjusted *p*-value: * ≤ 0.05, ** ≤ 0.01.

Pre-immunity to a historical H1N1 influenza virus was established in ferrets by intranasal inoculation of Sing/86 H1N1 influenza virus (Figure 1A), then these pre-immune ferrets were vaccinated after with NA protein vaccines. After infection with CA/09, pre-immune ferrets that were mock-vaccinated lost ~10% of their original body weight by day 5 p.i. (Figure 4A). There was little or no weight loss for pre-immune ferrets that were vaccinated with the N1-I COBRA NA, Viet/04 NA, and CA/09 NA vaccines. Pre-immune NA-vaccinated ferrets had an average clinical sign score of 0.5 (Figure 4B) while the pre-immune mock-vaccinated ferrets maintaining an average score of 1 for days 2 to 4 p.i. During infection, three pre-immune mock-vaccinated ferrets recovered, and one reached the clinical endpoint (Figure 4C). Pre-immune, mock-vaccinated ferrets had significantly higher viral nasal wash titers (1–2 log_10_ PFU/mL) compared to pre-immune NA-vaccinated ferrets, regardless of the NA vaccine (Figure 4D). There was no statistical difference in the nasal wash titers between any of the pre-immune, NA-vaccinated ferret groups. Viral nasal wash titers were undetectable by day 5 p.i.

### 3.3. N1-I COBRA NA Mitigated Clinical Signs in Naive Ferrets Directly Challenged with Viet/04 H5N1 Virus

**Figure 5:**
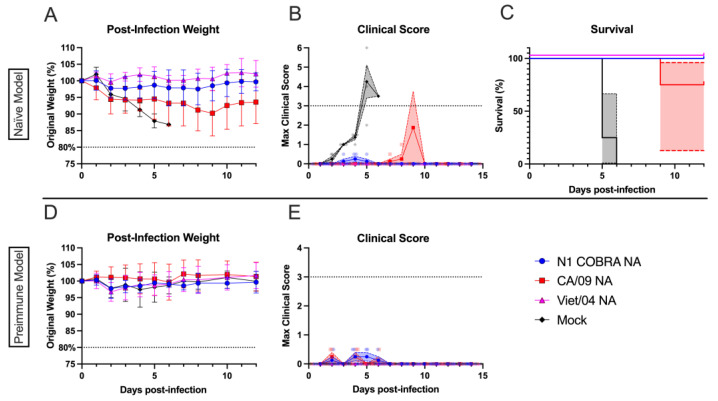
N1-I COBRA NA vaccine performed similarly to the Viet/04 NA vaccine in the naïve ferret model intranasally challenged with Viet/04 H5N1. (**A**) After infection with Viet/04, the weight during the infection was normalized to the percentage of the original starting weight before infection. (**B**) The maximum clinical scores from the morning and evening checks were displayed with a score of three or greater indicating clinical endpoint. Individual ferret clinical scores are shown with the line connecting the mean score and the range of the standard error of the mean shaded. (**C**) The survival percentage for naïve ferret model were determined for each vaccine group. The 95% asymmetrical confidence intervals are shown as shadow for the groups that did not have 100% survival. (**D**) Weights were also monitored for the Sing/86 H1N1 pre-immune group, in addition to the clinical signs. (**E**) Survival was 100% for all groups in the pre-immune vaccinated Viet/04 infection model. All error bars represent standard deviation. The minimum weight before clinical endpoint was 75%, with 80% denoting an increase in clinical scoring.

The efficacies of the vaccines were tested in the highly pathogenic Viet/04 virus infection model with naïve ferrets that were vaccinated with NA vaccines. Mock-vaccinated naïve ferrets had a continuous weight loss after infection, and all the ferrets reached clinical endpoint by day 6 p.i. (Figure 5A–C). Ferrets vaccinated with CA/09 NA vaccines lost ~5% of their original weight by day 4 p.i. with slow decline to 10% of their body weight by day 9 p.i. (Figure 5A). Ferrets vaccinated with the N1-I COBRA NA or the Viet/04 NA all survived infection with little to no weight loss or clinical signs over the 14 days of observation (Figure 5A–C). There were no viral nasal wash titers detected at any time point post-infection.

### 3.4. Pre-Immunity to Historical H1N1 Influenza Viruses Mitigated Clinical Signs and Mortality When Directly Challenged with Viet/04 H5N1 Influenza Virus Compared to Naïve Animals

Naïve ferrets that were initially pre-immunized with the Sing/86 H1N1 influenza virus, then vaccinated, and then challenged with Viet/04 virus had no weight loss, had minimal clinical signs, and no mortality over the course of the infection (Figure 5D–E). No viral nasal wash titers were detected over the course of infection. There was no distinguishable difference between pre-immune ferrets vaccinated with the N1-COBRA NA, CA/09 NA, or Viet/04 NA vaccines and pre-immune mock-vaccinated ferrets. When compared to the mock-vaccinated naïve ferret group (Figure 5A–C), the mock-vaccinated pre-immune ferrets (Figure 5D–E) had fewer clinical manifestations due to Sing/86 pre-immunity. 

### 3.5. N1-I COBRA NA Mitigated Viral Nasal Wash Titers in Naïve Ferrets Directly Challenged with Bris/07 H1N1

**Figure 6:**
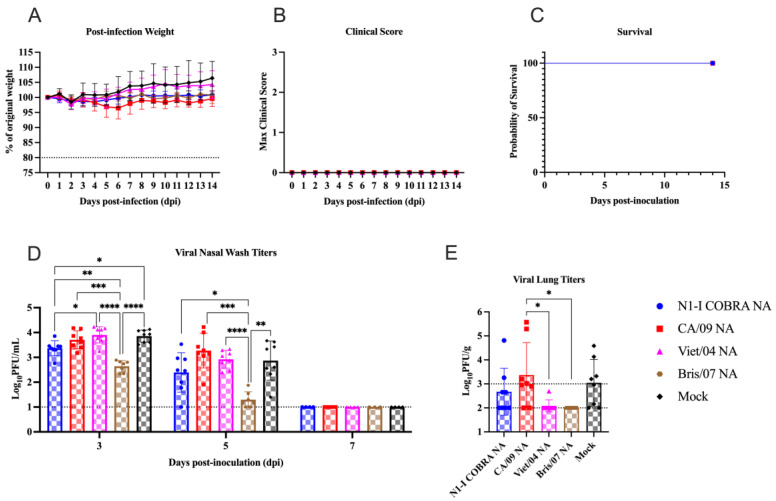
N1-I COBRA NA vaccine performed similarly to the Bris/07 NA vaccine in the naïve ferret model intranasally challenged with Bris/07 H1N1. (**A**) After infection with Bris/07, the weight during infection was normalized to the percentage of the original starting weight before infection. (**B**) The maximum clinical scores from the morning and evening checks were displayed with a score of three or greater indicating clinical endpoint. (**C**) Survival was determined after accounting for censored histopathology ferrets purposefully euthanized day 5 p.i. (**D**) The viral nasal wash titers for all vaccinated groups were determined through plaque assay and analyzed with a mixed effects model. (**E**) The viral lung titers were quantified by plaquing homogenized tissue each the upper and lower left lung lobes. The limit of detection (LOD; dashed line) was 1.0 log_10_(PFU/mL) and 2.0 log_10_(PFU/g) for the nasal wash titers and lung titers, respectively. The limit of quantification (dotted line) was 2.0 log_10_(PFU/mL) and 3.0 log_10_(PFU/g) for the nasal wash titers and lung titers, respectively. All error bars represent standard deviation. Adjusted *p*-value: * ≤ 0.05, ** ≤ 0.01, *** ≤ 0.001, **** ≤ 0.0001.

Naïve ferrets were vaccinated with the N1-I COBRA NA or one of the wild-type NA vaccines, then infected with the Bris/07 virus, had no weight loss or clinical signs and no mortality over the course of the infection (Figure 6A–C). Viral shedding patterns in nasal wash samples were investigated (Figure 6D) by plaque assay with MDCK cells. Ferrets from all groups shed virus in their nasal wash samples at day 3 p.i., and stop shedding by day 7 p.i. On day 3 p.i., N1-I COBRA NA and Bris/07 NA-vaccinated ferrets had significantly lower viral titers in their nasal wash samples than the mock-vaccinated ferrets, while CA/09 NA and Viet/04 NA-vaccinated ferrets had similar viral shedding level as the mock-vaccinated ferrets. By day 5 p.i., the Bris/07 NA-vaccinated ferrets had minimal viral shedding in their nasal wash samples, and at or near the limit of detection (1.0 log_10_(PFU/mL)), whereas the average of viral shedding of N1-I COBRA NA-vaccinated ferrets was lower than the ferrets in mock group, but not significantly different due to the large standard deviation. As contrast, the CA/09 NA and Viet/04 NA-vaccinated ferrets had similar or even higher viral shedding levels than the ferrets in mock group on day 5 p.i. Sections of lung lobes were collected on day 5 p.i. to determine the viral loads in ferret lungs (Figure 6E). The Bris/07 NA-vaccinated ferrets had undetectable viral titers in the lung sections, while the ferrets in all other four groups had detectable viral titers in the lung sections. The ferrets vaccinated with N1-I COBRA NA had a lower but not significant viral loads in the lung, compared to the ferrets in the mock group. The ferrets vaccinated with CA/09 NA vaccine had a similar viral titer as the ferrets in the mock group. The ferrets vaccinated with Viet/04 NA had a significant lower viral titer in the lung, compared to the ferrets in the mock group. 

### 3.6. Serological Responses following Vaccination

**Figure 7:**
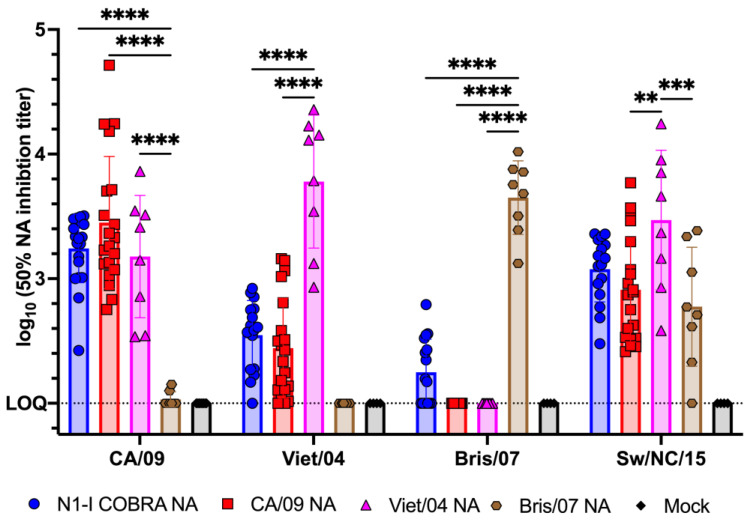
N1-I COBRA NA vaccine elicited NA-inhibiting antibodies to a panel of genetically diverse HXN1 viruses. The 50% NA inhibition (NAI) titers elicited by the NA-based vaccines were measured for a panel of N1 influenza viruses that belong to different N1 genetic clades. Non-linear regression of the ELLA results using RDE treated ferret sera was used to determine the 50% NAI titers (Supplementary Materials). The N1-I COBRA NA vaccination elicited inhibitory antibodies to viruses in the panel. The CA/09 NA and Viet/04 NA elicited cross-reactive NAI antibodies to each other. The Bris/07 NA vaccine elicited NAI antibodies to itself. All NA vaccines elicited antibodies that inhibited the SW/NC/15 virus. The initial serum dilution used for the ELLA assay (1:100) was defined as the limit of quantification at 2.0 log_10_ (50% NAI titer). All error bars represent standard deviation. Adjusted *p*-value: ** ≤ 0.01, *** ≤ 0.001, **** ≤ 0.0001.

Serum samples were collected from naive ferrets that were vaccinated with COBRA or wild type NA vaccines (Figure 1) and assayed for the elicitation of anti-NA antibodies with the ability to inhibit NA enzymatic activity against a panel of HxN1 viruses (Figure 7). The N1-I COBRA NA elicited antibodies with ELLA activity against all four viruses in the panel. Not all of the N1-I COBRA NA-vaccinated animals seroconverted to Bris/07 (Clade: N1.2). The CA/09 NA and Viet/04 NA-vaccinated animals only had antibodies with ELLA titers against the CA/09 (Clade: N1.1), Viet/04 (Clade: N1.1) and Sw/NC/15 (Clade: N1.3) viruses. The antibodies elicited by Bris/07 NA were even more restricted in breadth with ELLA titers against only the Bris/07 and SW/NC/15 viruses. 

Sera with HAI activity against CA/09 virus was detected in naïve ferrets vaccinated with CA/09 HA. The CA/09 HA vaccine-elicited titers ranged between 4.32 and 9.32 (log_2_ HAI titer), with a mean of 7.031 (log_2_ HAI titer). All ferrets pre-immunized with Sing/86 H1N1 seroconverted with an average log_2_ titer of 7.978 with a standard deviation of +/− 0.5453. Pre-immunized, mock-vaccinated ferrets had no antibodies with cross-reactive that inhibited either the CA/09 H1N1 or Viet/04 H5N1 HA activity. 

### 3.7. Contact Transmission from Naïve Ferrets That Were Vaccinated with HA or NA Vaccines to Unvaccinated Naïve Ferrets

**Figure 8:**
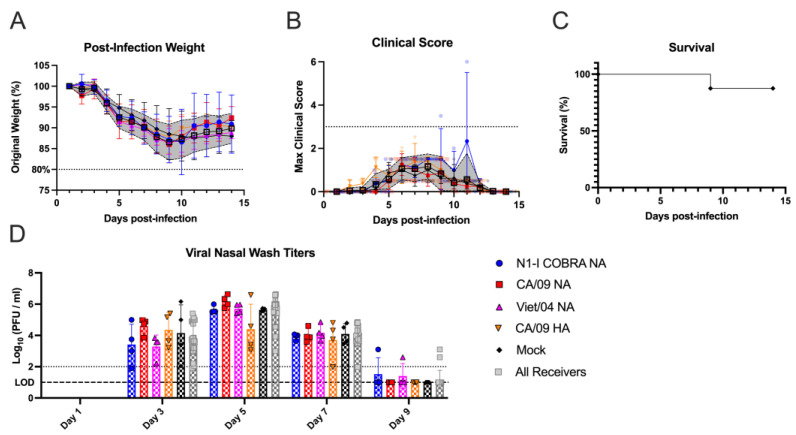
Vaccination of contact transmitting ferrets did not limit the transmission to other ferrets or affect the receiving ferret’s clinical or viral outcomes. Naïve ferrets were co-housed one day after the CA/09 intranasal infection of ferrets vaccinated with different influenza protein antigens (see legend; Figure 1B). (**A**) The normalized weights, regardless of the transmitting ferret’s vaccination status, were the not significantly different, and the combination of all the naïve receivers is shown in grey with the ‘All Receivers’ group. The clinical scores (**B**), and survival (**C**) were recorded over the course of the study and shown as All Receivers. (**D**) The viral lung titers determined through plaque assay were also unsignificant. The maximum clinical scores from the morning and evening checks were displayed with a score of three or greater indicating clinical endpoint. Individual ferret clinical scores are shown with the line connecting the mean score, and the individual points with lower opacity. The grey shaded regions for weight and clinical score indicate the standard error of all receivers combined. Mixed effects analysis or two-way ANOVA with repeated measures were used for statistical analysis. All error bars represent standard deviation. The minimum weight before clinical endpoint was 75%, with 80% denoting an increase in clinical scoring.

To determine if NA vaccination reduces the virus transmission from vaccinated animals to unvaccinated animals, unvaccinated naïve ferrets (Receiver) were co-housed with CA/09 influenza-virus infected HA- or NA-vaccinated ferrets (Transmitter) at day 1 p.i. (Figure 1B). Contact transmission occurred between all vaccinated transmitter and influenza-naïve receiver pairs. All naïve receiving ferrets lost body weight that was not statistically different compared to mock-vaccinated animals, regardless of the transmitting ferret’s vaccination (F-statistic: 0.1029; DF: 4, 15; *p*-value: 0.9797) (Figure 8A). Further, the HA- or NA-vaccination in transmitting ferrets did not significantly contribute to the naïve receiving ferrets’ clinical scores (F-statistic: 1.916; DF: 3, 12; *p*-value: 0.1809), or nasal wash titer (F-statistic: 2.549; DF: 4, 15; *p*-value: 0.0825) (Figure 8B,D). 

### 3.8. Contact Transmission from Unvaccinated Naïve Ferrets to Naïve Ferrets That Were Vaccinated with HA or NA Vaccines

**Figure 9:**
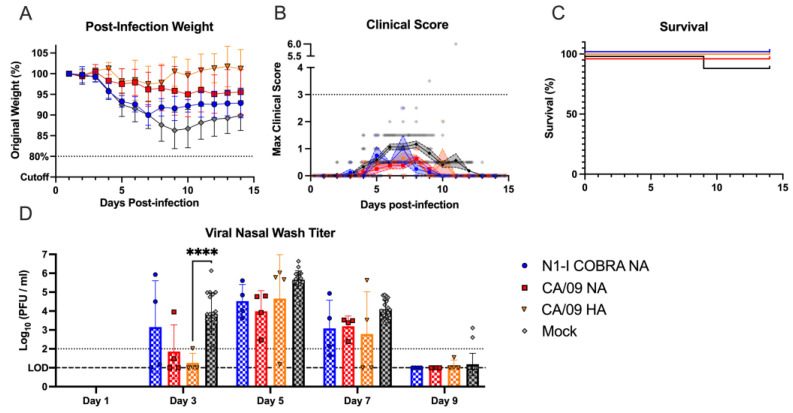
Vaccination of contact receiving ferrets did not limit transmission. (**A**) The weight during infection was normalized to the percentage of the original starting weight before co-housing on day 1 p.i. (**B**) The maximum clinical scores from the morning and evening checks were displayed with a score of three or greater indicating clinical endpoint. Individual ferret clinical scores are shown (lower opacity) with the line connecting the mean score (full opacity) and the standard error of the mean shaded. (**C**) The survival percentage for each group was determined. (**D**) The nasal wash titers for all vaccinated groups were determined through plaque assay. The limit of detection (LOD; dashed line) was 1.0 log_10_ (PFU/mL), and the limit of quantification (dotted line) was 2.0 log_10_ (PFU/mL). Mixed effects analysis with repeated measures was used for statistical analysis. Tukey’s test for multiple comparisons was used for the weight analysis, and Dunnett’s test was used for nasal wash titers with the mock receivers as the control group. All error bars represent standard deviation. The minimum weight before clinical endpoint was 75%, with 80% denoting an increase in clinical scoring. Adjusted *p*-value: **** ≤ 0.0001.

**Figure 10:**
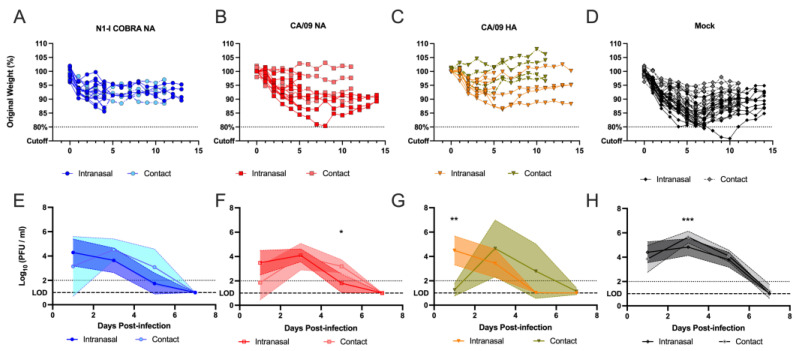
Magnitude of weight loss and viral loads did not change based upon intranasal or contact transmission inoculation method. Vaccinated animals that were challenged by either direct intranasal inoculation (intranasal) or through contact transmission from an intranasally inoculated cage mate (contact) were compared by offsetting the contact weights by three days and the nasal wash titers by two days. The offset days were chosen to maximize the alignment of the mock-vaccinated animals (**D**,**H**). Each individual ferret’s weight loss over time was depicted for each vaccine group (**A**–**D**). The average nasal wash titer with standard deviations were depicted for each vaccine group as well (**E**–**H**). Comparisons were conducted within vaccine groups with a two-way repeated measured ANOVA and Sidak’s multiple comparison test. The minimum weight before clinical endpoint was 75%, with 80% denoting an increase in clinical scoring. The limit of detection (LOD; dashed line) was 1.0 log_10_(PFU/mL), and the limit of quantification (dotted line) was 2.0 log_10_(PFU/mL). Adjusted *p*-value: * ≤ 0.05, ** ≤ 0.01, *** ≤ 0.001.

A second set of vaccinated ferrets were designated as the receiving ferrets and co-housed with unvaccinated naïve ferrets that were intranasally infected with CA/09 virus (Figure 1C). All the unvaccinated transmitting ferrets exhibited similar weight loss, clinical scores, and nasal wash titers amongst each other (Figure 2A–D). The mock-vaccinated receiving ferrets lost 15–20% of their body weight, whereas CA/09 HA-vaccinated receiving ferrets only lost marginal weight over the 14 days of co-housing (Figure 9A). In contrast, the N1-I COBRA NA-vaccinated receiving ferrets lost ~10% of their original body weight by day 7 p.i., which was not statistically different than the CA/09 NA-vaccinated receiving ferrets (Figure 9A). The CA/09 NA-vaccinated receiving ferrets initially maintained weight, but steadily lost weight over time, declining to an average of 5% of their original body weight. Two of the four CA/09 NA-vaccinated ferrets lost considerable weight compared to the other two ferrets that maintained their original weight during infection (Figure 10B). The weight of N1-I COBRA NA-vaccinated receiving ferrets was statistically different than CA/09 HA-vaccinated receiving ferrets on days 4, 9, and 12 p.i. Overall, the HA vaccine completely mitigated weight loss in receiving ferrets. The NA vaccines, including the N1-I COBRA NA, partially mitigated weight loss in receiving ferrets with few observed clinical signs (Figure 9A,B) and all vaccinated receiving ferrets survived the infection (Figure 9C). 

The viral nasal wash titers did not differ significantly between the HA- and NA-vaccinated receivers (Figure 9D). In comparison to the mock-vaccinated receiving ferrets, only the CA/09 HA-vaccinated receivers had significantly lower viral nasal wash titers on day 3 p.i. Contact transmission provided increased variability in the nasal wash titers on day 3 p.i. The standard deviation of the mock-vaccinated receivers ranged from 0.478 to 1.104 log_10_ (PFU/mL) between days 3 and 9 p.i. The N1-I COBRA NA-vaccinated receiving ferrets had a wide standard deviation of 2.453 PFU/mL on day 3 p.i., followed by more narrow intervals up to day 9 p.i. Viral shedding titers peaked for all groups of receiving ferrets at day 5 p.i. and then declined to undetectable levels on day 9 p.i. (Figure 9D). 

### 3.9. Comparison of the Inoculation Methods

The results from the two ferret transmission studies were compared to determine differences in vaccine effectiveness between the two routes of virus administration: intranasal and contact. The contact transmission weights were normalized by three days to match the intranasal inoculated ferret study (Figure 10A–D). The N1-I COBRA NA-vaccinated ferrets had similar weight loss profiles regardless of the inoculation method. Ferrets maintained between 90 and 97% of their original body weight (Figure 10A). The receiving ferrets that were infected by using the direct contact method had weight loss curves that were similar to the weights of transmitting ferrets directly receiving virus via the intranasal method. The CA/09 NA-vaccinated receiving ferrets lost less body weight than the intranasally inoculated transmitting ferrets, however these animals also had the largest variability (Figure 10B). Similar results were observed for CA/09 HA-vaccinated ferrets (Figure 10C). The mock-vaccinated ferrets had similar responses to both inoculation methods, but had greater variability with a range of peak weight loss (80–95%) (some animals reached clinical endpoint at bodyweight 80% from the resulting increase in clinical score) (Figure 10D).

The nasal wash titers from intranasal and contact transmission were also compared by offsetting the contact groups by two days (Figure 10E–H). The mean peak viral titer did not differ between the intranasal and contact methods in any of the vaccine groups. The mock-vaccinated receiving ferrets had a significantly higher peak titer compared to the intranasal inoculated ferrets (adj. *p*-value: 0.0001). Although all groups reached a peak titer between 4 to 6 log_10_ (PFU/mL), the viral dynamics differed between the groups of different vaccines. For instance, in the CA/09 NA vaccine groups, ferrets that were infected by intranasal inoculation had significantly lower viral shedding titers on the fifth day compared to ferrets that were infected by contacting with infected animals (Figure 10F). In the CA/09 HA-vaccine groups, ferrets that were infected by contacting with infected animals had a delay in the increase in viral shedding titers (Figure 10G), but still reached the same viral peak titer observed on day 3 p.i. for the intranasal group.

## 4. Discussion

Antibodies elicited against the influenza NA protein is associated with decreased influenza H1N1 shedding and illness in humans [22]. Therefore, the N1-I COBRA NA was investigated for its potential as an influenza vaccine antigen. The naïve ferret model was used to thoroughly characterize the protective responses elicited by the N1-I COBRA NA after infection. The N1-I COBRA NA induced similar ferret responses and viral titers compared to the CA/09 HA and NA positive controls. The Bris/07 NA vaccine was included as a heterologous NA and does not elicit NAI antibodies to either the H1N1 or H5N1 viruses. The ferrets in N1-I COBRA NA and positive control vaccine groups consistently had lower mean viral titers in nasal washes and lung tissue compared to ferrets in both Bris/07 NA and mock vaccine groups. Although differences in the mean weight loss compared to ferrets in other vaccine groups were similar, only 50% of the Bris/07 NA-vaccinated ferrets survived infection. The severity and distribution of inflammation (histopathological scores) in the vaccinated animals were similar in the ear and nasal cavity. Even CA/09 HA-vaccinated ferrets, which had undetectable viral titers in both the nasal wash and lung tissue, the inflammation levels were similar to the ferrets in all other groups infected with CA/09 influenza virus. The N1-I COBRA NA vaccine reduced inflammation in ferrets as the CA/09 NA vaccine, but not as much as the CA/09 HA vaccine. These results correlate with the presence of virus still in the lungs in the NA-vaccinated animals on day 5 p.i., while the viral loads for the ferrets in CA/09 HA group was below the limit of detection. 

The N1-I COBRA NA vaccine provided protection against seasonal and pandemic N1 viruses. The N1-I COBRA NA vaccine elicited antibodies with lower NAI titers than antibodies elicited by the Viet/04 NA, but both equally protected ferrets against Viet/04 virus infection. Therefore, the NAI titers and magnitude of protection against the H5N1 virus may not be directly correlated. There, potentially, is a minimum NAI antibody titer threshold for protection. Furthermore, ferrets vaccinated with the N1-I COBRA NA vaccine had little weight loss or mortality compared to the CA/09 NA-vaccinated ferrets over the course of infection.

The protective responses in ferrets corresponded with the elicited serological responses. The N1-I COBRA NA vaccine elicited strong responses against both CA/09 and Viet/04 viruses in 100% of the ferrets/group. The viral inhibition titer was lower against Bris/07, but was similar against the Sw/NC/15. The wildtype NA vaccines had antigenic profiles similar to their specific lineages, as previously observed [16]. One contrasting observation was that the Bris/07 NA vaccine elicited antibodies that inhibited the NA of Sw/NC/15. Previously in the mouse model, there was no cross-reaction between clades (N1.2 and N1.3) [16]. This difference in specificity may be due to the change in animal models, from mice to ferrets. 

The majority of the human population has pre-existing immunity to influenza through both vaccination and infection. This pre-existing immunity biases the immune response recall response and is termed immune imprinting [23,24,25,26,27,28,29]. The N1-I COBRA NA was tested in a pre-immune ferret model to mimic individuals exposed to H1N1 influenza viruses. The N1-I COBRA NA vaccine performed equally well, as the CA/09 NA-vaccinated control ferrets in both the naïve and pre-immune models. Therefore, even with the pre-existing anti-Sing/86 N1 NA antibodies, a protective response was elicited when vaccinated with the N1-I COBRA NA. This effect was prominent in the CA/09 virus infected ferrets. Following the Viet/04 virus infection in pre-immune ferrets, there was no weight loss for any group of ferrets. Contrary to expectations, the pre-immunity elicited by the Sing/86 H1N1 virus was more protective against the Viet/04 H5N1 infection than in the CA/09 H1N1 challenge. It was expected that the Sing/86 pre-immunity would be more protective in the CA/09 infection than in the Viet/04 infection because both Sing/86 and CA/09 are H1N1 subtype viruses. Since, the serum from Sing/86 pre-immunized ferrets did not have HAI activity for either of the viruses (all HAI titers were less than 1:10), the differences in protection may be a results of stem binding antibodies or T-cell responses induced from pre-immunization with live virus infection. 

Vaccination should not only protect the individuals who are vaccinated, but also nearby associated people. In the contact transmission model, the receiving ferrets were co-housed with the transmitting ferrets for the entirety of the observation period. Our results indicated that vaccination with either NA- or HA-based vaccines did not inhibit the contact transmission by the CA/09 virus. This was previously observed in the pig model as well [30]. This transmission model mimicked family transmission between individuals who are frequently in contact. One of the limitations of this model is that it does not capture shorter exposure periods. Since the viral dynamics differed in the vaccinated groups, it may suggest varying windows for transmission post-infection. 

In addition, the viral dynamics after aerosol transmission in vaccinated ferrets may also differ due to the differences in inoculum particulate size. Additionally, inflammatory responses were only measured on day 5 p.i., that may be informative for collective lung tissue, but may have been past the time point to observe significant quantitative nasal cavity inflammation. Earlier time points of the upper respiratory tract may have provided differential results when comparing vaccine groups. Lastly, within the pre-immune ferret model, the pre-existing antibodies to the HA protein of Sing/86 virus may interfere with the measurement of the functional NA-specific antibodies and prohibit comparison between the elicited NA antibodies in both naïve and pre-immune models. 

Overall, N1-I COBRA NA is a promising candidate for a broadly protective influenza vaccine. Inclusion of the N1-I COBRA NA can enhance current split-inactivated vaccines or be included in new influenza vaccine formulations. Split-inactivated vaccines elicit mostly an HA-specific antibody response with minimal NA response. Inclusion of the broadly protective NA antigens, such as the N1-I COBRA NA, opens the door to eliciting a more balanced response after vaccination. New influenza vaccine candidates, whether subunit or microparticles, can also be designed to include the N1-I COBRA NA. The N1-I COBRA NA can help us achieve the future of the influenza vaccines eliciting a broadly protective response to multiple antigens and increasing the potential protection that individuals receive.

## Data Availability

The data are contained within the article and supplemental materials.

## Supplementary Materials

The following supporting information can be downloaded at: https://www.mdpi.com/article/10.3390/v15010184/s1, Figure S1. Coomassie staining for the purified NA proteins; Figure S2. NA Protein sequences alignment. The NA catalytic site residues are in red boxes, while the framework residues are in blue boxes.

## References

[1] Philippon D.A.M., Wu P., Cowling B.J., Lau E.H.Y. (2020). Avian Influenza Human Infections at the Human-Animal Interface. J. Infect. Dis..

[2] Levine M.Z., Holiday C., Jefferson S., Gross F.L., Liu F., Li S., Friel D., Boutet P., Innis B.L., Mallett C.P. (2019). Heterologous prime-boost with A(H5N1) pandemic influenza vaccines induces broader cross-clade antibody responses than homologous prime-boost. NPJ Vaccines.

[3] Jang Y.H., Seong B.L. (2019). The Quest for a Truly Universal Influenza Vaccine. Front. Cell. Infect. Microbiol..

[4] Krammer F., Weir J.P., Engelhardt O., Katz J.M., Cox R.J. (2020). Meeting report and review: Immunological assays and correlates of protection for next-generation influenza vaccines. Influ. Other Respir. Viruses.

[5] Music N., Tzeng W.P., Liaini Gross F., Levine M.Z., Xu X., Shieh W.J., Tumpey T.M., Katz J.M., York I.A. (2019). Repeated vaccination against matched H3N2 influenza virus gives less protection than single vaccination in ferrets. NPJ Vaccines.

[6] Ferdinands J.M., Thompson M.G., Blanton L., Spencer S., Grant L., Fry A.M. (2021). Does influenza vaccination attenuate the severity of breakthrough infections? A narrative review and recommendations for further research. Vaccine.

[7] Choi A., Ibanez L.I., Strohmeier S., Krammer F., Garcia-Sastre A., Schotsaert M. (2020). Non-sterilizing, Infection-Permissive Vaccination With Inactivated Influenza Virus Vaccine Reshapes Subsequent Virus Infection-Induced Protective Heterosubtypic Immunity From Cellular to Humoral Cross-Reactive Immune Responses. Front. Immunol..

[8] Johansson B.E., Moran T.M., Kilbourne E.D. (1987). Antigen-presenting B cells and helper T cells cooperatively mediate intravirionic antigenic competition between influenza A virus surface glycoproteins. Proc. Natl. Acad. Sci. USA.

[9] Zheng A., Sun W., Xiong X., Freyn A.W., Peukes J., Strohmeier S., Nachbagauer R., Briggs J.A.G., Krammer F., Palese P. (2020). Enhancing Neuraminidase Immunogenicity of Influenza A Viruses by Rewiring RNA Packaging Signals. J. Virol..

[10] Krammer F., Fouchier R.A.M., Eichelberger M.C., Webby R.J., Shaw-Saliba K., Wan H., Wilson P.C., Compans R.W., Skountzou I., Monto A.S. (2018). NAction! How Can Neuraminidase-Based Immunity Contribute to Better Influenza Virus Vaccines?. mBio.

[11] Wohlbold T.J., Nachbagauer R., Xu H., Tan G.S., Hirsh A., Brokstad K.A., Cox R.J., Palese P., Krammer F. (2015). Vaccination with adjuvanted recombinant neuraminidase induces broad heterologous, but not heterosubtypic, cross-protection against influenza virus infection in mice. MBio.

[12] Smith G.E., Sun X., Bai Y., Liu Y.V., Massare M.J., Pearce M.B., Belser J.A., Maines T.R., Creager H.M., Glenn G.M. (2017). Neuraminidase-based recombinant virus-like particles protect against lethal avian influenza A(H5N1) virus infection in ferrets. Virology.

[13] Chen Y.Q., Wohlbold T.J., Zheng N.Y., Huang M., Huang Y., Neu K.E., Lee J., Wan H., Rojas K.T., Kirkpatrick E. (2018). Influenza Infection in Humans Induces Broadly Cross-Reactive and Protective Neuraminidase-Reactive Antibodies. Cell.

[14] Couch R.B., Atmar R.L., Franco L.M., Quarles J.M., Wells J., Arden N., Nino D., Belmont J.W. (2013). Antibody correlates and predictors of immunity to naturally occurring influenza in humans and the importance of antibody to the neuraminidase. J. Infect. Dis..

[15] Monto A.S., Petrie J.G., Cross R.T., Johnson E., Liu M., Zhong W., Levine M., Katz J.M., Ohmit S.E. (2015). Antibody to Influenza Virus Neuraminidase: An Independent Correlate of Protection. J. Infect. Dis..

[16] Skarlupka A.L., Bebin-Blackwell A.G., Sumner S.F., Ross T.M. (2021). Universal influenza virus neuraminidase vaccine elicits protective immune responses against human seasonal and pre-pandemic strains. J. Virol..

[17] Giles B.M., Ross T.M. (2011). A computationally optimized broadly reactive antigen (COBRA) based H5N1 VLP vaccine elicits broadly reactive antibodies in mice and ferrets. Vaccine.

[18] Roubidoux E.K., Schultz-Cherry S. (2021). Animal Models Utilized for the Development of Influenza Virus Vaccines. Vaccines.

[19] Skarlupka A.L., Ross T.M. (2020). Immune Imprinting in the Influenza Ferret Model. Vaccines.

[20] Ecker J.W., Kirchenbaum G.A., Pierce S.R., Skarlupka A.L., Abreu R.B., Cooper R.E., Taylor-Mulneix D., Ross T.M., Sautto G.A. (2020). High-Yield Expression and Purification of Recombinant Influenza Virus Proteins from Stably-Transfected Mammalian Cell Lines. Vaccines.

[21] Skarlupka A.L., Ross T.M. (2021). Inherent Serum Inhibition of Influenza Virus Neuraminidases. Front. Vet. Sci..

[22] Maier H.E., Nachbagauer R., Kuan G., Ng S., Lopez R., Sanchez N., Stadlbauer D., Gresh L., Schiller A., Rajabhathor A. (2020). Pre-existing Antineuraminidase Antibodies Are Associated With Shortened Duration of Influenza A(H1N1)pdm Virus Shedding and Illness in Naturally Infected Adults. Clin. Infect. Dis. Off. Publ. Infect. Dis. Soc. Am..

[23] Gostic K.M., Ambrose M., Worobey M., Lloyd-Smith J.O. (2016). Potent protection against H5N1 and H7N9 influenza via childhood hemagglutinin imprinting. Science.

[24] Tesini B.L., Kanagaiah P., Wang J., Hahn M., Halliley J.L., Chaves F.A., Nguyen P.Q.T., Nogales A., DeDiego M.L., Anderson C.S. (2019). Broad Hemagglutinin-Specific Memory B Cell Expansion by Seasonal Influenza Virus Infection Reflects Early-Life Imprinting and Adaptation to the Infecting Virus. J. Virol..

[25] Worobey M., Han G.Z., Rambaut A. (2014). Genesis and pathogenesis of the 1918 pandemic H1N1 influenza A virus. Proc. Natl. Acad. Sci. USA.

[26] Miller M.S., Gardner T.J., Krammer F., Aguado L.C., Tortorella D., Basler C.F., Palese P. (2013). Neutralizing antibodies against previously encountered influenza virus strains increase over time: A longitudinal analysis. Sci. Transl. Med..

[27] Hancock K., Veguilla V., Lu X., Zhong W., Butler E.N., Sun H., Liu F., Dong L., DeVos J.R., Gargiullo P.M. (2009). Cross-reactive antibody responses to the 2009 pandemic H1N1 influenza virus. N. Engl. J. Med..

[28] Lessler J., Riley S., Read J.M., Wang S., Zhu H., Smith G.J., Guan Y., Jiang C.Q., Cummings D.A. (2012). Evidence for antigenic seniority in influenza A (H3N2) antibody responses in southern China. PLoS Pathog..

[29] Monsalvo A.C., Batalle J.P., Lopez M.F., Krause J.C., Klemenc J., Hernandez J.Z., Maskin B., Bugna J., Rubinstein C., Aguilar L. (2011). Severe pandemic 2009 H1N1 influenza disease due to pathogenic immune complexes. Nat. Med..

[30] Everett H.E., van Diemen P.M., Aramouni M., Ramsay A., Coward V.J., Pavot V., Canini L., Holzer B., Morgan S., Dynamics sLoLa C. (2021). Vaccines That Reduce Viral Shedding Do Not Prevent Transmission of H1N1 Pandemic 2009 Swine Influenza A Virus Infection to Unvaccinated Pigs. J. Virol..

