# Supplementary material for: Multi-Influenza HA Subtype Protection of Ferrets Vaccinated with an N1 COBRA-Based Neuraminidase
Source: Viruses. 2023 Jan 9;15(1):184. doi: 10.3390/v15010184 (PMC9865009; doi:10.3390/v15010184)
Supplement: Supplementary file 1 [file viruses-15-00184-s001.zip › viruses-2108206-supplementary.pdf]

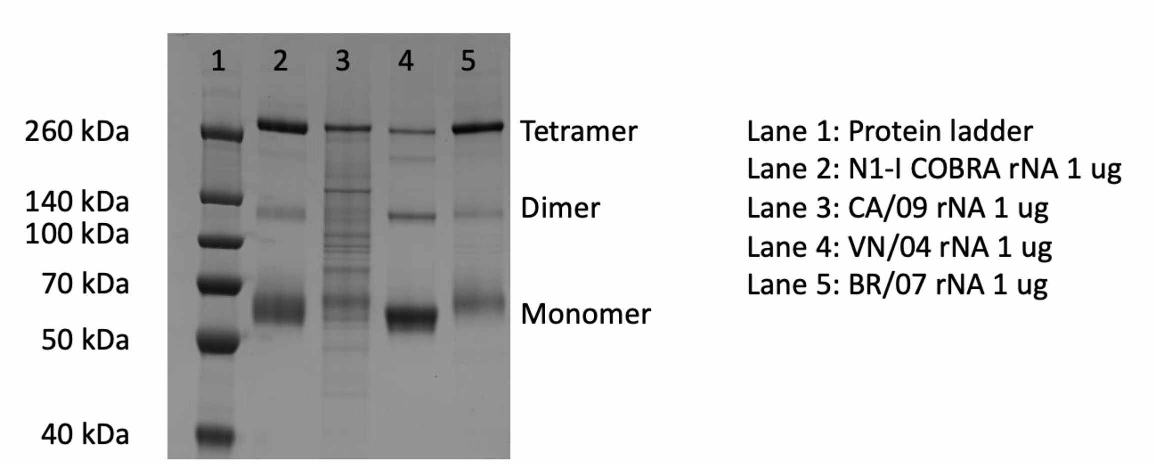

Figure S1. Coomassie staining for the purified NA proteins.

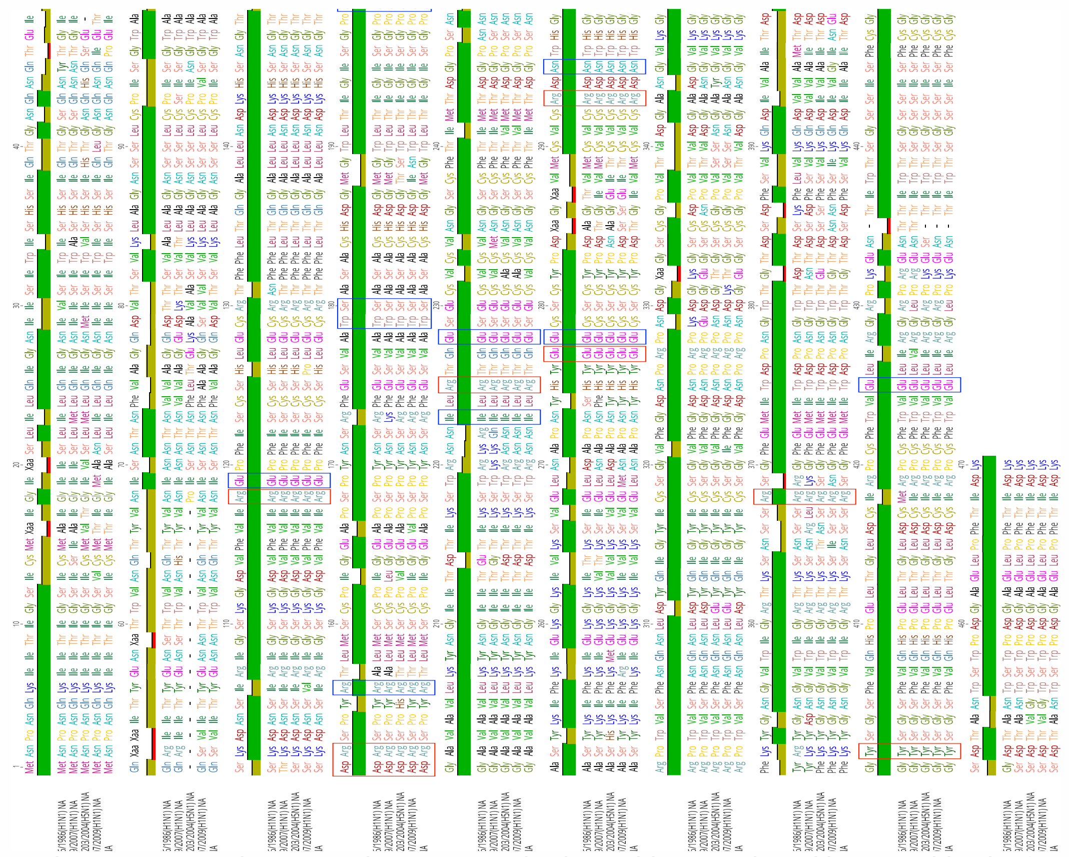

Figure S2. NA Protein sequences alignment. The NA catalytic site residues are in red boxes, while the framework residues are in blue boxes.
